# Diagnosis of unexposed tumours using endobronchial ultrasonography with a guide sheath and a thin transbronchial needle

**DOI:** 10.1002/rcr2.713

**Published:** 2021-01-25

**Authors:** Haruka Kuno, Rei Sainouchi, Takayuki Simamoto, Aya Miyagawa‐Hayashino, Yoshizumi Takemura

**Affiliations:** ^1^ Department of Pulmonary Medicine Kyoto Kuramaguchi Medical Center Kyoto Japan; ^2^ Department of Surgical Pathology Kyoto Prefectural University of Medicine Kyoto Japan

**Keywords:** Endobronchial ultrasonography with the guide sheath, transbronchial biopsy, transbronchial needle aspiration

## Abstract

Transbronchial diagnosis of unexposed lung tumours is challenging in clinical practice. Although modified transbronchial needle aspiration (TBNA) is used for this purpose, the diagnostic yield is unsatisfactory. In such cases, conventional endobronchial ultrasonography with a guide sheath and transbronchial biopsy (TBB) is also ineffective. We found TBB was feasible by placing a guide sheath with a thin transbronchial needle into the tumours. We report two cases of unexposed tumours diagnosed successfully with this technique. Case 1 presents a typical carcinoid in the peripheral lung. Case 2 presents a squamous cell carcinoma at the third bifurcation of the right lung. TBB samples obtained this way were larger than TBNA samples. Moreover, multiple TBBs were possible once the guide sheath was inserted intratumourally. In the modern era of precision medicine, larger amounts of tissues are required for multiple downstream analyses. This novel technique will make a significant contribution towards diagnosing unexposed lung tumours.

## Graphical abstract

We report two cases of unexposed tumours diagnosed successfully using transbronchial biopsy (TBB) done by placing a guide sheath with a thin transbronchial needle into the tumours . Case 1 presents a typical carcinoid in the peripheral lung. Case 2 presents a squamous cell carcinoma at the third bifurcation of the right lung. TBB samples obtained this way were larger than transbronchial needle aspiration (TBNA) samples. Moreover, multiple TBBs were possible once the guide sheath was inserted intratumorally. This novel technique will make a significant contribution towards diagnosing unexposed lung tumours.

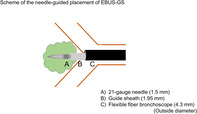

## Introduction

**Figure 1:**
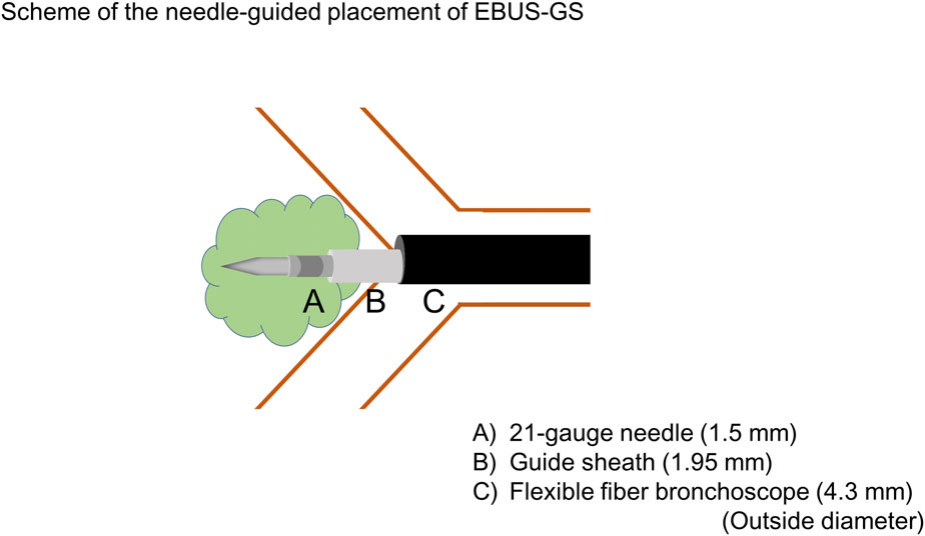
Scheme of the needle‐guided placement of endobronchial ultrasonography with a guide sheath. (A) 21‐Gauge needle, 1.5 mm in outside diameter (PeriView FLEX; Olympus Ltd). (B) Guide sheath, 1.95 mm in outside diameter (SG‐200C; Olympus Ltd). (C) Flexible fibre bronchoscope, 4.3 mm in outside diameter (BF‐P290; Olympus Ltd).

Transbronchial biopsy (TBB) is not feasible for tumours located behind the bronchial wall. Although transbronchial needle aspiration (TBNA) may be performed, the diagnostic yield is low. The recently released PeriView FLEX (Olympus Ltd, Tokyo, Japan), a 21‐gauge transbronchial needle, is thin enough to enter in a guide sheath of 1.95 mm in diameter and can be placed into the tumours through the bronchial wall. We performed endobronchial ultrasonography with the guide sheath (EBUS‐GS) for obtaining TBB for such extrinsic tumours. Here, we report two cases diagnosed successfully using this novel sampling technique. Figure 1 demonstrates the scheme of the needle‐guided placement of EBUS‐GS.

## Case Report

### Case 1

**Figure 2:**
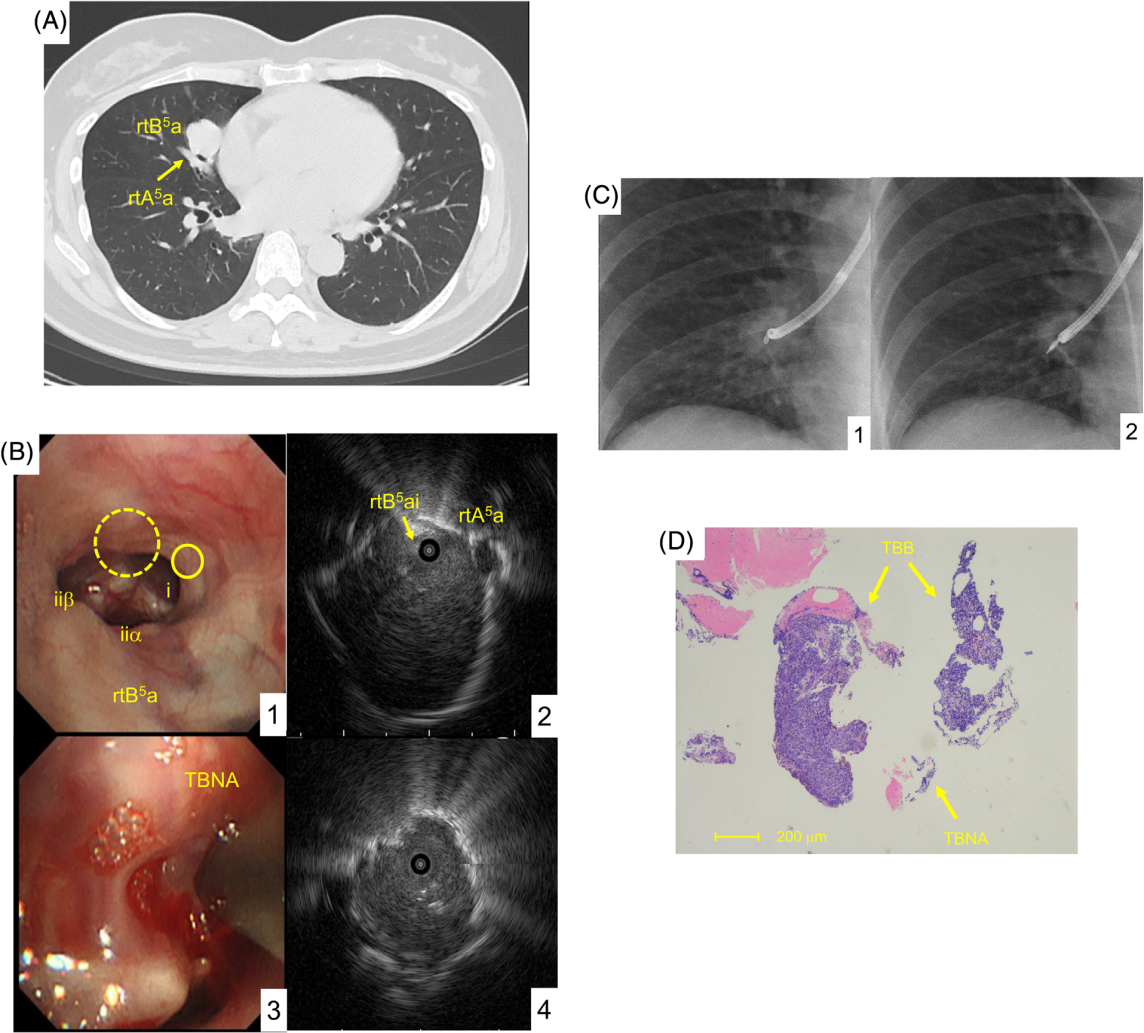
Case 1. (A) Chest computed tomography. A round‐shaped tumour (25 mm in size) at the right segment 5 was observed in front of the right bronchus (rtB5a) and pulmonary artery (rtA5). (B) The images of bronchoscopic examination. (1) The bronchus was intact. The tumour was suggested to be on the other side of the bifurcation at the 12 o'clock position (circled by a dashed line). The pulmonary artery is circled by a solid line. (2) Adjacent tumour echo and pulmonary artery (rtA5a) dorsal to the tumour was observed from the entry at rtB5ai by endobronchial ultrasonography (EBUS). (3) Overview of transbronchial needle aspiration (TBNA). (4) “Within” tumour echo was observed by EBUS through indwelled guide sheath. X‐ray image is shown in C2. (C) X‐ray images. (1) After TBNAs, guide sheath was intratumourally placed. (2) EBUS was performed through indwelled guide sheath. (D) The overview of the specimen obtained by needle aspiration and the forceps (haematoxylin–eosin staining, 4×).

A 46‐year‐old woman with no smoking history was referred to our hospital for evaluation of a chest radiography abnormality. The patient was asymptomatic, with no significant physical and laboratory findings. However, chest computed tomography (CT) revealed a rounded mass, 25 mm in diameter, in segment (S)5 in the right (rt) middle lobe (Fig. 2A). Bronchoscopy revealed intact bronchus and bifurcation of rt bronchus (B)5ai, rtB5aiiα, and rtB5aiiβ (Fig. 2B1). Before needling, we located the tumour and the right pulmonary artery (rtA5a) to ascertain the puncture position (Fig. 2B2). After two successful TBNAs (Fig. 2B3), we delivered the guide sheath into the tumour (Figs 1, 2), detected the tumour as “within” echo by EBUS (Fig. 2B4, C2), and conducted forceps biopsies. The biopsy specimens obtained were larger than in conventional TBNAs (Fig. 2D). Immunohistochemical staining revealed chromogranin A, CD56, and synaptophysin‐positive cells in both the aspirate and the biopsies; however, the biopsies revealed only 2% Ki‐67 labelling index. Consequently, the patient was diagnosed with typical carcinoid, a low‐grade neuroendocrine tumour, and underwent radical surgery.

### Case 2

**Figure 3:**
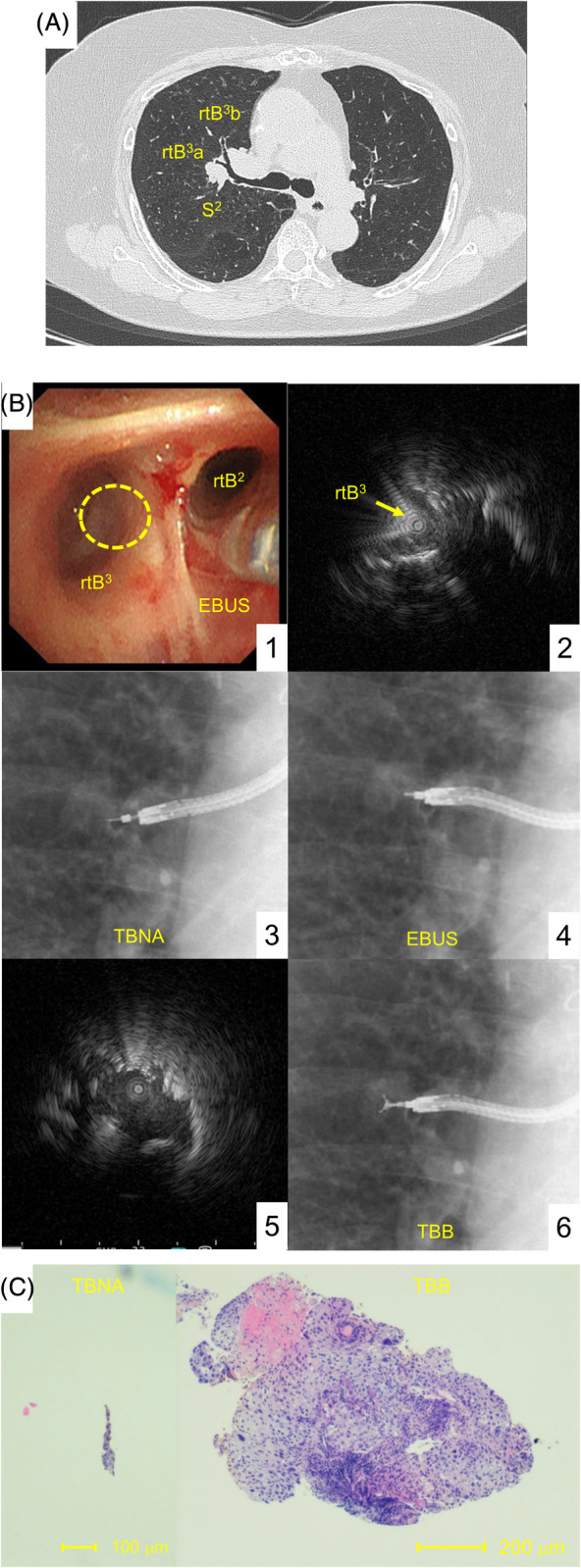
Case 2. (A) Chest computed tomography. A border irregular tumour (25 mm in size) at the bifurcation of right bronchi (rtB2) and (rtB3) was observed. (B) (1) The view of bronchoscopic examination at the bifurcation of rtB2 and rtB3. The bifurcation was intact. The suggested area of the target is circled using a dashed line. (2) Adjacent tumour echo was observed from the entry at rtB3 by endobronchial ultrasonography (EBUS). (3) X‐ray image of transbronchial needle aspiration (TBNA). (4) X‐ray image of EBUS through intratumourally placed guide sheath. (5) “Within” tumour echo was observed. (6) Lastly, transbronchial biopsies (TBBs) were conducted. (C) The overview of the specimen obtained by TBNA (left side) and TBB (right side). (haematoxylin–eosin staining, 4×).

A 68‐year‐old woman, treated for asthma and chronic obstructive pulmonary disease overlap at our clinic, was referred to our hospital. The patient had undergone surgery for resection of a brachiocephalic artery aneurysm at a neighbouring hospital a year ago. Her CT revealed an irregular tumour, 25 mm in diameter, at the bifurcation of rtB2 and rtB3 (Fig. 3A). Bronchoscopy ensured the intactness of the bifurcation and the bronchus wall where convex probe EBUS could not reach (Fig. 3B1). Once we ascertained the tumour location and ensured no major vessel involvement by EBUS (Fig. 3B1, B2), we needled twice (Fig. 3B3). Next, we delivered the guide sheath into the tumour, detected the tumour as within echo by EBUS (Fig. 3B4, B5), performed forceps biopsies (Fig. 3B6), and obtained bigger tissue. Although no tumour cells were identified in one of the TBNA specimens, the patient was diagnosed with squamous cell carcinoma (Fig. 3C) and underwent chemoradiotherapy.

## Discussion

In the early 2000s, conventional TBNA was introduced, mainly to diagnose bulky mediastinal lymph nodes. Needling was done at the expected tumour location based on CT images, as little information was available from inside the lung. After two decades, we can now needle a broad area of the lung. We manipulate EBUS‐TBNA for central tumours as far as the convex probe can reach or perform TBNA for the remaining peripheral tumours. However, despite improved access, the diagnostic yields of TBNAs do not fulfil clinical needs. Naso et al. reported that needle aspiration had a limited diagnostic yield of ~70% for malignancies, and only 69% of positive specimens contained adequate volume for molecular genetic testing [1].

In contrast, EBUS‐GS TBB provides promising sensitivity and specimen volume when it detects the target as within echo. Furthermore, once the guide sheath is placed, TBB can be repeated. However, EBUS‐GS is ineffective if it cannot reach the target and create an echo image [2]. Thus, both TBNA and EBUS‐GS TBB have different characteristics in terms of accessibility and stability.

The new aspiration needle, PeriView FLEX, is thinner than conventional needles and can be installed in the guide sheath with a 1.95‐mm outside diameter. Originally designed for diagnosing peripheral tumours, we found this needle works as a guide for the guide sheath to go through the bronchial wall smoothly, not only through peripheral bronchus but also through the central thick bronchial wall, such as the third bifurcation as shown in case 2. We speculate that this is due to thin diameter of the guide sheath and the marginal difference between outer diameters of the needle and the guide sheath.

Takai et al. reported a trial for peripheral targets [3]. They conducted TBNA through a guide sheath after EBUS, achieving a diagnostic yield of 86.5%. Subsequently, Arimura et al. reported a similar trial, performing transbronchial brushing and TBB, with diagnostic yields of 62.9%, 77.1%, and 80.0% for brushing, TBNA, and TBB, respectively [4]. Furthermore, Huang et al. showed a novel TBNA technique for central targets arranged by EBUS survey to ensure the puncture location, resulting in 87.5% diagnostic yield [5]. However, all these trials were performed with conventional (thick) devices, without delivering a guide sheath into tumours, and TBB or TBNA was performed after EBUS. The present study reports the first two cases on transbronchial needle‐guided EBUS‐GS placement and biopsies for extrinsic tumours.

We cannot draw clear conclusions on the indication and management of complications; however, we would like to suggest our perspectives. First, the indicated bronchus for needle placement should be observable under bronchoscopy as the only way of holding the needle at the right position is by pushing and fixing the tip of the fibrescope against the bronchus; otherwise, the needle, together with the bronchoscope, moves smoothly as far as the pleura. Second, the management of complications, especially bleeding, is important and can be achieved using three‐dimensional CT or virtual bronchoscopy. In addition, we conducted an EBUS survey before needling to know the location of tumours and major vessels. Finally, we delivered the guide sheath after successful needle aspirations.

In conclusion, transbronchial needle (PeriView FLEX)‐guided EBUS‐GS and biopsies for extrinsic tumours were practical and provided an adequate specimen for diagnosis and molecular testing. Further studies are needed before establishing this approach for clinical use.

### Disclosure Statement

Appropriate written informed consent was obtained for publication of this case report and accompanying images.

### Author Contribution Statement

Haruka Kuno and Takayuki Simamoto were the attending physicians of case 1. Rei Sainouchi was the attending physician of case 2. Yoshizumi Takemura designated and directed this project. Aya Miyagawa‐Hayashino made pathological diagnosis of both cases.

## References

[1] Naso J , Bras J , Villamil C , et al. 2020 Cytologic features and diagnostic value of PeriView FLEX transbronchial needle aspiration targeting pulmonary nodules. Cancer Cytopathol. 128:333–340. 31995670 10.1002/cncy.22240

[2] Okachi S , Imai N , Imaizumi K , et al. 2016 Factors affecting the diagnostic yield of transbronchial biopsy using endobronchial ultrasonography with a guide sheath in peripheral lung cancer. Intern. Med. 55:1705–1712. 27374669 10.2169/internalmedicine.55.6341

[3] Takai M , Izumo T , Chavez C , et al. 2014 Transbronchial needle aspiration through a guide sheath with endobronchial ultrasonography (GS‐TBNA) for peripheral pulmonary lesions. Ann. Thorac. Cardiovasc. Surg. 20:19–25. 24807472 10.5761/atcs.oa.13-00261

[4] Arimura K , Sekine Y , Hiroshima K , et al. 2017 The efficacy of transbronchial needle aspiration with endobronchial ultrasonography using a guide sheath for peripheral pulmonary lesions suspected to be lung cancer. Respir. Investig. 55:365–371. 10.1016/j.resinv.2017.08.004 29153417

[5] Huang Z , Huang H , Ning Y , et al. 2019 Radial probe endobronchial ultrasound assisted conventional transbronchial needle aspiration in the diagnosis of solitary peribronchial pulmonary lesion located in the segmental bronchi. J. Cancer 10:634–642. 30719161 10.7150/jca.28755 PMC6360410

